# An Interpretable Hierarchical Belief Rule Base for the Performance Evaluation of Laser Inertial Measurement Units

**DOI:** 10.3390/e28101048

**Published:** 2026-09-24

**Authors:** Yongjia Gao, Jieyu Liu, Shuanzhu Li, Can Li, Qiang Shen, Zhaoqiang Wang

**Affiliations:** 1 College of Missile Engineering, PLA Rocket Force University of Engineering, Xi’an 710025, China; 2 Shandong Simanke Technology Co., Ltd., Jining 272500, China

**Keywords:** laser strapdown inertial measurement unit, hierarchical belief rule base, Bayesian inference, information entropy, interpretability optimization

## Abstract

Laser strapdown inertial measurement units (LIMUs) are critical components of autonomous navigation and attitude determination systems used in mission-critical platforms such as spacecraft. Their performance directly affects the reliability and safety of space missions. To address the challenges posed by high-dimensional indicator sets, limited data, and stringent interpretability requirements, this paper proposes an interpretability-aware hierarchical Bayesian belief rule base model, termed IH-BRB, for LIMU performance evaluation. First, a five-level hierarchical evaluation structure is developed based on the physical architecture and error-propagation relationships of a LIMU, thereby reducing rule base complexity and improving inference traceability. Second, an interlayer transmission mechanism based on the expected grade scores and entropy-derived reliability is introduced to preserve essential uncertainty information and dynamically adjust the attribute weights of upper-level BRB nodes. Third, semantic consistency constraints are imposed on the conditional probability tables and rule-consequent belief distributions to prevent semantic drift during data-driven optimization. The proposed model is validated using an engineering LIMU dataset with expert-defined target grades to assess its consistency with the established grading criterion. The results show that IH-BRB maintains a competitive evaluation performance while preserving physically meaningful rule semantics and layer-wise traceability.

## 1. Introduction

LIMUs offer advantages such as continuous navigation outputs, manageable manufacturing costs, and a high degree of autonomy, and have therefore been widely used in space missions involving launch vehicles and spacecraft [1,2,3]. However, prolonged storage may lead to gradual degradation in the LIMU performance. Once the degradation reaches a critical level, it may seriously compromise the safety of missions such as crewed spaceflight and satellite launches, potentially causing substantial economic losses and endangering personnel [4]. Therefore, research into LIMU performance evaluation provides a theoretical basis for selecting the most suitable LIMU before critical space missions and is of considerable importance for improving mission reliability.

Current approaches for the performance evaluation of complex systems can be broadly categorized into three types: knowledge-based methods, data-driven methods, and knowledge–data hybrid-driven methods. Among them, evaluation methods that rely on domain knowledge primarily depend on experts’ understanding of the system structure, operational mechanisms, and failure patterns, and are typically implemented using tools such as analytic hierarchy process (AHP) [5], fuzzy comprehensive evaluation [6], fault tree analysis [7], and Bayesian networks [8] to conduct qualitative or semi-quantitative assessments of the system performance. Such methods offer favorable interpretability and engineering practicality, and are capable of adequately reflecting the interrelationships among system components and the logical correlations among various indicators. However, due to their heavy dependence on expert experience during the modeling process, these methods are susceptible to subjective biases that may lead to deviated evaluation results, and they also fall short in accurately characterizing the nonlinear coupling relationships among indicators in complex systems.

Data-driven evaluation methods, in contrast, are approaches that leverage historical samples and employ models such as machine learning [9], support vector machines [10], random forests [11], and neural networks [12] to extract the latent mapping relationships between the input indicators and system performance levels from data. These methods exhibit favorable nonlinear and adaptive learning capabilities, and can achieve high evaluation accuracy when sufficient samples are available. However, their drawback lies in the general requirement for a large amount of high-quality sample data to support model training. For certain engineering problems with high costs, failure samples and degradation samples are often difficult to obtain in adequate quantities for training purposes. Moreover, some data-driven models possess “black-box” characteristics, which render them incapable of explaining the rationale behind the evaluation results, thereby hampering engineering personnel in fault tracing and decision-making analysis.

Knowledge–data hybrid-driven evaluation methods integrate the strengths of both knowledge-driven and data-driven approaches. On one hand, they employ expert knowledge to establish evaluation indicator systems, rule structures, or even prior models, thereby endowing the evaluation process with a clear physical meaning and logical rationale; on the other hand, they update and optimize model parameters through learning from data, thus enhancing the model’s adaptability to actual sample distributions and complex nonlinear relationships. Typical examples include belief rule base [13], fuzzy neural networks [14], and Bayesian network-based evaluation models combined with data learning [15]. Compared with purely knowledge-driven methods, such approaches can mitigate the influence of subjective experience on evaluation results, while, relative to purely data-driven methods, they are able to preserve a certain degree of interpretability and engineering traceability.

The belief rule base (BRB) model was formally proposed by Yang et al. [16] in 2006. It can embed expert knowledge in the form of if–then rules, represent uncertainty through consequent belief distributions, and optimize model parameters using sample data, thereby possessing both interpretability and adaptive capability. Due to the limited sample size and the strong requirement for engineering interpretability in LIMU performance evaluation, BRB provides a suitable framework for integrating expert knowledge with observed data. However, when a conventional BRB is directly applied to high-dimensional inputs, the number of rules increases rapidly with the number of attributes and reference levels, leading to rule explosion [17].

To mitigate the rule explosion problem, several approaches have been proposed. Disjunctive BRBs avoid exhaustive combinations of attribute reference values [18], while attribute-selection-based ensemble BRBs reduce the dimensionality of individual rule bases [19]. Approximate BRBs further simplify the rule structure by constructing rules from individual attributes [20]. Hierarchical and multilayer BRB models decompose a high-dimensional problem into a set of lower-dimensional sub-BRBs, thereby reducing the combinatorial growth of the rule base [21,22]. Despite their effectiveness in reducing model complexity, attribute reduction may discard useful information, simplified rule structures may reduce the explicit representation of multi-attribute interactions, and the effectiveness of hierarchical models depends on how the attributes are organized.

For LIMU performance evaluation, hierarchical modeling is more suitable for retaining the complete indicator information while controlling the rule scale. However, complexity reduction alone does not ensure model interpretability. The hierarchical structure should have clear engineering semantics, while interlayer transmission needs to retain essential uncertainty information without introducing excessive rule complexity. In addition, data-driven optimization should preserve the semantic consistency of the rule base [23]. These considerations lead to three key issues addressed in this study: hierarchical structure interpretability, interpretable interlayer information transmission, and semantic consistency during parameter optimization.

The remainder of this paper is organized as follows: Section 2 presents the problem description; Section 3 constructs the interpretable hierarchical Bayesian BRB model; Section 4 designs the interpretable optimization method; Section 5 conducts experimental verification and result analysis; and Section 6 concludes the paper.

## 2. Problem Formulation

The core of BRB is a set of “if–then” rules, each associated with a belief structure. For a BRB model with *d* input attributes, *N* output evaluation levels, and *R* rules, the *r*-th rule is generally expressed as follows:

(1) $$\begin{array}{cc} R_{r}:\; & \mathrm{IF}\;x_{1}\;\mathrm{is}\;A_{1,j_{r,1}}\wedge x_{2}\;\mathrm{is}\;A_{2,j_{r,2}}\wedge \cdots \wedge x_{d}\;\mathrm{is}\;A_{d,j_{r,d}}\\ \; & \mathrm{Then}\;\left\{\left(D_{1},\beta_{r,1}\right),\left(D_{2},\beta_{r,2}\right),\cdots ,\left(D_{N},\beta_{r,N}\right)\right\}\\ \; & \mathrm{with}\;\mathrm{Rule}\;\mathrm{weight}\;\theta_{r}\;\mathrm{and}\;\mathrm{Attribute}\;\mathrm{weight}\;\delta_{i} \end{array}$$

where $x_{i}$ denotes the *i*-th input attribute; $A_{i,j_{r,i}}$ is the $j_{r,i}$-th reference level corresponding to the *i*-th attribute; $D_{n}$ is the *n*-th output evaluation grade; and $\beta_{r,n}$ denotes the degree of support assigned by the consequent of the *r*-th rule to $D_{n}$. The consequent belief distribution $\left\{\left(D_{1},\beta_{r,1}\right),\ldots ,\left(D_{N},\beta_{r,N}\right)\right\}$ allows the support of a rule to be distributed among multiple possible output grades, thereby representing the uncertainty contained in the rule conclusion.

Since a LIMU consists of multiple precision inertial devices, its performance evaluation requires the consideration of multiple underlying indicators. If all these indicators are directly used as inputs to a conventional BRB model, the numbers of rules and consequent belief-degree parameters increase exponentially as the input dimensionality increases, as follows:

(2) $$R_{\mathrm{flat}}=\prod_{i=1}^{d}J_{i},\;P_{\mathrm{flat}}=R_{\mathrm{flat}}N$$

where $J_{i}$ denotes the number of reference values for the *i*-th input attribute, and $R_{\mathrm{flat}}$ and $P_{\mathrm{flat}}$ denote the total number of rules and the total number of consequent belief-degree parameters in the conventional BRB model, respectively.

For domain experts, specifying the consequent belief degrees for every rule in such a large-scale rule base based on expert knowledge is extremely difficult. Therefore, directly constructing a conventional BRB model is unsuitable for interpretable LIMU performance evaluation. To alleviate rule base and parameter explosion, this paper adopts a hierarchical BRB modeling strategy that partitions the high-dimensional indicator set among several low-dimensional BRB nodes according to the physical structure of the LIMU. Suppose that the hierarchical model consists of $N_{B}$ BRB nodes, where the *l*-th node contains $d_{l}$ input attributes and its *i*-th input attribute has $J_{l,i}$ reference levels. The total number of rules in the hierarchical model is then given by the following:

(3) $$R_{HBRB}=\sum_{l=1}^{N_{B}}\prod_{i=1}^{d_{l}}J_{l,i}$$

Since the input dimension $d_{l}$ of each sub-node is much smaller than the original input dimension *d*, the hierarchical structure can decompose the global high-dimensional rule space into multiple low-dimensional local rule spaces, thereby significantly reducing the scale of the rule base. However, the hierarchical BRB model cannot solely aim at compressing the number of rules. For LIMU performance evaluation, hierarchical modeling also needs to simultaneously satisfy three requirements, namely, structural interpretability, interlayer propagation interpretability, and local inference interpretability, so as to provide decision-makers with a traceable evaluation structure. Based on the above analysis, this paper focuses on addressing the following three problems.

Problem 1: Physical interpretability of indicator selection and hierarchical structure.

LIMU performance evaluation involves multiple dimensions of underlying performance indicators. The way in which hierarchical partitioning is carried out with respect to indicator selection directly determines the engineering semantics and inference traceability of the model. If a data-driven approach is adopted to partition the hierarchical structure, it may result in issues such as unclear physical meanings of the nodes, ambiguous attribution of indicators, and unexplainable inference paths. Therefore, it is necessary to select key performance degradation indicators based on the physical composition, functional hierarchy, and error propagation relationships of the LIMU, and to establish an interpretable hierarchical BRB structure in which each node within every layer and the propagation relationships between layers possess clear engineering significance, thereby ensuring the traceability of the overall model.

Problem 2: Interpretability of interlayer information propagation.

The hierarchical BRB model needs to propagate inference results between different layers. If the complete belief distribution is directly propagated, the uncertainty information from the lower-level nodes can be preserved; however, this will increase the input dimensionality of the upper-level nodes, causing the rule explosion problem to shift from the lower layers to the upper layers. Conversely, if only a single utility value is propagated, the credibility and uncertainty information embedded in the lower-level inference results will be compressed and lost. Therefore, a low-dimensional interlayer transmission mechanism is required to retain essential performance and uncertainty information without increasing the scale of upper-level rule bases.

Problem 3: Interpretability of local BRB optimization.

The rule consequents and optimized parameters of local BRB nodes should conform to the degradation laws of LIMU performance. During parameter optimization, if only fitting accuracy is taken into account, the resulting rule consequent distributions may deviate from engineering cognition, thereby undermining the semantic consistency of the rules. Therefore, probability validity, engineering monotonicity, and semantic consistency constraints should be imposed during optimization to preserve the interpretability of the optimized rule base.

## 3. Interpretable Hierarchical Bayesian Belief Rule Base Model

### 3.1. Interpretable Hierarchical BRB Structure Design

#### 3.1.1. LIMU Evaluation Indicator Selection

The reasonableness of the performance evaluation indicator system determines whether the inference results of the model possess engineering significance. For the purpose of interpretable performance evaluation of a LIMU, the selection of indicators needs to satisfy three principles: (1) mechanism representativeness, meaning that the selected indicators should be capable of representing the core error sources along the error propagation chain of the inertial measurement unit and should directly reflect the degree of performance degradation of the devices; (2) engineering measurability, meaning that these indicators should be common assessment items in ground calibration tests and periodic inspections, with stable and reliable measurement means available; and (3) hierarchical partitionability, meaning that the indicator system should have a clear physical attribution hierarchy to support the establishment of a hierarchical inference model.

In the performance testing of a LIMU, a primarily self-calibration-based approach is generally adopted. Various parameters acquired during the testing process can reflect the performance information of the inertial devices. Therefore, this paper selects bias stability, scale factor stability, and installation errors to construct the underlying indicator system. Since the laser strapdown inertial measurement unit consists of three laser gyroscopes and three accelerometers, the three laser gyroscopes and three accelerometers are denoted by $G_{x}$, $G_{y}$, $G_{z}$, $A_{x}$, $A_{y}$, and $A_{z}$, respectively, to facilitate subsequent modeling. For each sensitive axis, two orthogonal installation error indicators, one bias stability indicator, and one scale factor stability indicator are selected as the performance indicators for that axis. Consequently, the six sensitive axes constitute a 24-dimensional underlying input vector (6×4=24):

(4) $$\begin{array}{cc} X= & \{M_{G_{xy}},M_{G_{xz}},M_{G_{yx}},M_{G_{yz}},M_{G_{zx}},M_{G_{zy}},M_{A_{xy}},M_{A_{xz}},M_{A_{yx}},M_{A_{yz}},M_{A_{zx}},M_{A_{zy}},\\ \; & B_{G_{x}},B_{G_{y}},B_{G_{z}},B_{A_{x}},B_{A_{y}},B_{A_{z}},K_{G_{x}},K_{G_{y}},K_{G_{z}},K_{A_{x}},K_{A_{y}},K_{A_{z}}\} \end{array}$$

To facilitate intuitive understanding of the physical organization of the 24-dimensional input vector, Figure 1 illustrates the correspondence between the LIMU structure, the six sensitive axes, and the associated underlying performance indicators.

As shown in Figure 1, the 24 input indicators are organized according to the six sensitive axes of the LIMU, with each axis corresponding to two installation error indicators, one bias stability indicator, and one scale factor stability indicator. Among these indicators, the installation error $M_{ab}$ is used to characterize the deviation of the sensitive axis from the ideal orthogonal coordinate system. It primarily represents the axial non-orthogonality error arising from device assembly processes and is generally expressed in arcminutes (′). The bias stability $B_{a}$ refers to the fluctuation of the bias over time during a single long-term power-on cycle of the device under no external input or under a constant input. It is one of the key parameters for characterizing the low-frequency drift properties of inertial devices. Its unit is °/h for gyroscopes and *g* for accelerometers. The scale factor stability $K_{a}$ is used to describe the long-term variation characteristics of the proportional coefficient between the input physical quantity and the output signal, and reflects the stability of the output response gain of the device.

#### 3.1.2. Hierarchical Structure of the LIMU

To meet the requirements of hierarchical interpretability and result traceability in LIMU performance evaluation, this paper constructs a five-level hierarchical belief rule base inference model for comprehensive LIMU performance assessment, following the actual physical composition logic from the single-axis device level through the subsystem level to the overall system level. The overall system performance is achieved by fusing the underlying indicator information level by level. The structure and functions of each level are defined as follows:

Level 1: Indicator input layer. This layer inputs the 24 underlying performance indicators corresponding to the six sensitive axes of the LIMU. Serving as the foundation of the model, it provides raw data inputs for subsequent hierarchical inference.

Level 2: Installation error inference layer. Considering that installation errors reflect the degree of non-orthogonality of the sensitive axes, an independent installation error BRB node is constructed for each sensitive axis. The two installation error parameters of the corresponding axis serve as inputs, and the comprehensive installation error status of that axis is derived accordingly.

Level 3: Axis-level performance fusion layer. This layer takes a single axis as the basic evaluation unit. The comprehensive installation error status, together with the bias stability $B_{a}$ and scale factor stability $K_{a}$ of that axis, is fed into the axis-level BRB node, which performs multisource information fusion within the axis and outputs the comprehensive performance status of the single axis, reflecting the degradation degree of the single-axis device.

Level 4: Subsystem-level performance fusion layer. According to differences in the types of the core sensitive devices in the LIMU, this layer further partitions the axis-level performance statuses into the gyroscope subsystem performance status and the accelerometer subsystem performance status, which respectively characterize the comprehensive health levels of the angular motion measurement unit and the linear acceleration measurement unit, thereby ensuring that the model evaluation results are consistent with the internal functional modules of the LIMU.

Level 5: Overall system evaluation layer. This layer takes the gyroscope subsystem performance status and the accelerometer subsystem performance status as inputs to the top-level BRB node, performs comprehensive performance fusion inference of the subsystems, and finally outputs the overall LIMU performance grade and its corresponding belief distribution.

In summary, the constructed five-level hierarchical BRB inference structure is consistent with the physical composition relationships of the LIMU. The inputs and outputs of each layer in the model correspond to clear engineering objects and physical meanings. This design not only avoids the lack of interpretability caused by directly performing black-box fusion on high-dimensional underlying indicators but also enables traceable analysis of the evaluation process through layer-by-layer belief-state propagation. The specific structure is illustrated in Figure 2.

To further clarify the rule-building procedure of the BRB nodes shown in Figure 2, the installation error BRB of the $G_{x}$-axis gyroscope is taken as an example. This node contains two input attributes, $M_{Gxy}$ and $M_{Gxz}$, which represent the two orthogonal installation error components associated with the $G_{x}$ axis. For rule construction, each input attribute is described by three ordered reference levels, namely Small (S), Medium (Me), and Large (L). Therefore, the antecedent combinations are obtained from the Cartesian product {S,Me,L}×{S,Me,L}, resulting in 3×3=9 belief rules. The complete rule base is listed in Table 1.

In Table 1, *E*, *G*, *M*, and *P* denote the four output performance grades, and $\beta_{r,n}$ represents the belief degree assigned by the *r*-th rule to the *n*-th output grade. The two input attributes are associated with attribute weights $\delta_{1}$ and $\delta_{2}$, respectively, while each rule is assigned a rule weight $\theta_{r}$. Thus, the antecedent part of the rule base is completely determined by the Cartesian combinations of the input reference levels. In the proposed IH-BRB, the consequent belief degrees are not independently assigned to each rule; instead, they are generated from the output-grade prior probabilities and attribute conditional probabilities through the Bayesian rule-generation mechanism described in Section 3.2.1. The remaining local BRB nodes in the hierarchical model are constructed following the same principle.

### 3.2. IH-BRB Model Inference

Building upon the constructed five-level hierarchical structure of the LIMU, this paper further establishes a complete inference process that integrates rule base generation based on Bayesian inference, local node BRB reasoning, and interlayer propagation based on utility values and information entropy reliability.

#### 3.2.1. Rule Base Generation Based on Bayesian Inference

The rule base serves as the primary knowledge source for inference and decision-making in the BRB model. The information contained therein—namely, the correspondence between the states of input attributes and the associated output evaluation grades—determines the model’s knowledge representation capability and inference credibility. Moreover, the number of parameters in the model affects the complexity and consistency of expert knowledge embedding, as well as the ex ante interpretability of the model [24]. To address this, the present study employs a Bayesian inference-based approach to generate the rule consequents. By mapping expert knowledge through the prior probabilities of output grades and the conditional probability tables of input attributes, the proposed approach obtains the consequent belief distribution for each rule, thereby reducing the number of parameters to be specified and alleviating the difficulty of rule base construction.

Consider an arbitrary BRB node with *d* input attributes. The reference-level set of the *i*-th attribute is denoted by $\left\{A_{i,1},A_{i,2},\ldots ,A_{i,J_{i}}\right\}$, and the output performance-grade set is denoted by $\left\{D_{1},D_{2},\ldots ,D_{N}\right\}$. To generate the rule consequent belief degrees, the output prior probability $\pi_{n}=P\left(D_{n}\right)$ is defined as the prior probability that the output grade is $D_{n}$. The set of prior probabilities reflects the overall distribution of the output grades and satisfies $\sum_{n=1}^{N}\pi_{n}=1$ and $\pi_{n}\geq 0$. The input conditional probability $p_{j,n}^{\left(i\right)}=P\left(A_{i,j}|D_{n}\right)$ denotes the probability that the *i*-th input attribute falls into the *j*-th reference level $A_{i,j}$ given the output performance grade $D_{n}$. It reflects the association between the input reference levels and the output performance grades, and satisfies $\sum_{j=1}^{J_{i}}p_{j,n}^{\left(i\right)}=1$ and $p_{j,n}^{\left(i\right)}\geq 0$. Consequently, the probability table for this BRB node can be obtained as shown in Table 2.

For a rule $R_{r}=\left\{A_{1,j_{r,1}},A_{2,j_{r,2}},\ldots ,A_{d,j_{r,d}}\right\}$, under the assumption that the input attributes are conditionally independent given the output grade, the consequent belief degree of this rule assigned to the output grade $D_{n}$ is given by Bayes’ theorem as follows:

(5) $$\beta_{r,n}=\frac{P\left(D_{n}\right)\prod_{i=1}^{d}P\left(A_{i,j_{r,i}}|D_{n}\right)}{\sum_{m=1}^{N}P\left(D_{m}\right)\prod_{i=1}^{d}P\left(A_{i,j_{r,i}}|D_{m}\right)}=\frac{\pi_{n}\prod_{i=1}^{d}p_{j_{r,i},n}^{\left(i\right)}}{\sum_{m=1}^{N}\pi_{m}\prod_{i=1}^{d}p_{j_{r,i},m}^{\left(i\right)}}$$

From Equation (5), it can be seen that the consequent belief degree $\beta_{r,n}$ of rule $R_{r}$ is expressed as a posterior probability jointly derived from the output prior probabilities and the conditional probabilities of the input attributes, representing the support of the rule for output grade $D_{n}$. The initial prior probabilities and conditional probability tables are specified by multiple domain experts based on engineering experience. Consequently, experts are only required to specify these probabilistic parameters, without the need to assign consequent belief degrees to each output grade for every rule individually. Accordingly, the number of consequent parameters is reduced from $R_{\mathrm{flat}}\times N$ in the conventional form to the following:

(6) $$N+\sum_{i=1}^{d}J_{i}N$$

Therefore, this method transforms the rule-level consequent parameters into attribute-level probabilistic parameters, thereby significantly reducing the cost of expert knowledge embedding and improving the reliability of the initial knowledge.

#### 3.2.2. BRB Node Inference

To match continuous inputs with discrete rule antecedents, the original indicators are first mapped to the preset reference levels. Suppose that the input $x_{i}$ lies between the adjacent reference values $A_{i}^{v}$ and $A_{i}^{\left(v+1\right)}$. A triangular linear membership function is employed to calculate the matching degrees with respect to the reference levels as follows:

(7) $$\alpha_{ij}=\left\{\begin{array}{c} \frac{A_{i}^{v+1}-x_{i}}{A_{i}^{v+1}-A_{i}^{v}}\;\;j=v\;\\ \frac{x_{i}-A_{i}^{v}}{A_{i}^{v+1}-A_{i}^{v}}\;\;j=v+1\\ 0\;\;\;\;\;\begin{array}{c} \;j=1,2,\ldots ,J_{i};\\ \;j\neq v;j\neq v+1 \end{array}\; \end{array}\right.$$

where $\alpha_{i,j}$ is the matching degree of the *i*-th indicator with respect to the *j*-th reference value; $J_{i}$ represents the number of reference values for the *i*-th indicator. The above mapping allows a smooth transition of continuous inputs from one level to another.

Upon obtaining the matching degrees of all input attributes, the activation weight of each rule is calculated by incorporating the rule weights, attribute weights, and antecedent matching degrees. The activation weight of the *r*-th rule, which represents the degree to which the rule supports the current sample, can be expressed as follows:

(8) $${\tilde{\omega }}_{r}=\theta_{r}\prod_{i=1}^{d}{\left(\alpha_{i,j_{r,i}}\right)}^{{\bar{\delta }}_{i}},\;{\bar{\delta }}_{i}=\frac{\delta_{i}}{\max_{s}\left(\delta_{s}\right)},\;\omega_{r}=\frac{{\tilde{\omega }}_{r}}{\sum_{s=1}^{R}{\tilde{\omega }}_{s}}$$

Subsequently, the evidential reasoning (ER) algorithm is adopted to fuse the consequent belief distributions of all activated rules. First, the activation weight and consequent belief degrees of the *r*-th rule are transformed into basic probability masses as follows:

(9) $$\begin{array}{c} m_{r,n}=\omega_{r}\beta_{r,n},\;n=1,2,\cdots ,N\\ m_{r,D}=1-\sum_{n=1}^{N}m_{r,n}=1-\omega_{r}\sum_{n=1}^{N}\beta_{r,n} \end{array}$$

where $m_{r,n}$ denotes the basic probability mass assigned to the evaluation grade $D_{n}$ by the *r*-th rule and $m_{r,D}$ denotes the global ignorance probability mass not assigned to any specific evaluation grade. For multiple activated rules, an iterative fusion formula is employed to combine them:

(10) $$\begin{array}{c} m_{n,\mathrm{br}(r)}=\lambda_{\mathrm{br}(r)}\left[m_{n,\mathrm{br}(r-1)}m_{r,n}+m_{n,\mathrm{br}(r-1)}m_{r,D}+m_{D,\mathrm{br}(r-1)}m_{r,n}\right]\\ m_{D,\mathrm{br}(r)}=\lambda_{\mathrm{br}(r)}m_{D,\mathrm{br}(r-1)}m_{r,D}\\ \lambda_{\mathrm{br}(r)}={\left[1-\sum_{n=1}^{N}\sum_{\begin{array}{c} t=1\\ t\neq n \end{array}}^{N}m_{n,\mathrm{br}(r-1)}m_{r,t}\right]}^{-1} \end{array}$$

where $m_{n,\mathrm{br}(r)}$ denotes the basic probability mass after fusing the first *r* rules, and $\lambda_{\mathrm{br}(r)}$ is the normalization coefficient that ensures the fused results satisfy the constraint that the sum of probabilities equals 1. After the fusion of all *R* rules, the final belief degree of each performance grade is given by the following:

(11) $${\hat{\beta }}_{n}=\frac{m_{n,br(R)}}{1-m_{D,br(R)}}n=1,2,\ldots ,N$$

Therefore, the output belief distribution of the local BRB node is given by the following:

(12) $$y=\{\left(D_{1},{\hat{\beta }}_{1}\right),\left(D_{2},{\hat{\beta }}_{2}\right),\ldots ,\left(D_{N},{\hat{\beta }}_{N}\right)\}$$

To obtain a scalar result that facilitates interlayer propagation and quantitative comparison, let the utility of performance grade $D_{n}$ be denoted by $u(D_{n})$. The overall utility of the node output is then expressed as follows:

(13) $$u\left(y\right)=\sum_{n=1}^{N}u\left(D_{n}\right){\hat{\beta }}_{n}$$

where u(y) denotes the quantitative utility value of the overall system performance obtained through inference.

#### 3.2.3. Interlayer Propagation Mechanism Based on Information Entropy Reliability and Utility Values

The way in which information propagates among different layers in a hierarchical structure is another important issue affecting the model performance and interpretability. The majority of previous works use utility values to represent the information transferred between layers. Although such compression of the input dimensionality reduces the model complexity, it does so at the cost of losing the uncertainty information contained in the lower-level inference results. For example, suppose that the utilities of the evaluation grades are set to [1, 2, 3, 4]. The two belief distributions [0.1, 0.3, 0.3, 0.3] and [0, 0.2, 0.8, 0] have the same expected utility value (i.e., 2.8), while their concentrations and uncertainties differ greatly. Thus, using utility values to propagate information makes it impossible for the upper-level nodes to distinguish whether a utility value corresponds to high confidence or high ambiguity, which undermines the rationality and reliability of interlayer fusion. On the other hand, if we directly propagate the complete output belief distribution of a lower-level node to the upper layer, most of the uncertainty information contained in the inference result can be preserved. However, this will dramatically increase the input dimensionality of the upper-level node, resulting in exponential growth in the number of rule combinations and thereby transferring the rule explosion problem from the lower layers to the upper ones.

In order to solve these aforementioned problems, we propose in this paper an interlayer propagation mechanism that is based on utility values and entropy-derived reliability. We use the utility value to represent the performance level of the lower-level node and reliability to quantify the credibility of its inference results, thereby reducing the dimensionality of interlayer transmission while preserving sufficient uncertainty information.

Suppose that the output belief distribution of a given BRB node is denoted by $\hat{\beta }=\left\{{\hat{\beta }}_{1},{\hat{\beta }}_{2},\ldots ,{\hat{\beta }}_{N}\right\}$. To measure the degree of uncertainty embedded in the output belief distribution, this paper adopts the normalized Shannon entropy, defined as follows:

(14) $$H\left(\hat{\beta }\right)=-\frac{1}{\ln N}\sum_{n=1}^{N}\hat{\beta_{n}}\ln\left(\hat{\beta_{n}}\right)$$

where *N* is the number of evaluation grades and lnN serves as the normalization factor to constrain the information entropy $H(\hat{\beta })$ within the range of [0,1]. When $\hat{\beta_{n}}=0$, it is stipulated that $\hat{\beta_{n}}\ln\hat{\beta_{n}}=0$. On this basis, an entropy-derived relative reliability coefficient for the node output is defined as follows:

(15) $$\rho =\frac{1}{1+H\left(\hat{\beta }\right)}$$

Because the normalized information entropy satisfies $H\left(\hat{\beta }\right)\in \left[0,1\right]$, the reliability coefficient is bounded within 0.5≤ρ≤1 and decreases monotonically as the normalized information entropy increases. When the output belief distribution is fully concentrated on a single evaluation grade, the normalized information entropy approaches 0 and ρ approaches 1, indicating that the node provides a relatively definite grade-oriented inference result. Conversely, when the output belief distribution approaches a uniform distribution, the normalized information entropy approaches 1 and ρ reaches its lower bound of 0.5, indicating substantial ambiguity among the evaluation grades. Nevertheless, the lower-level output is attenuated rather than completely discarded during interlayer propagation, because its expected utility still retains aggregated information about the overall performance position.

To incorporate the entropy-derived reliability coefficient into the upper-level BRB inference process, this paper constructs a sample-adaptive dynamic attribute-weight mechanism. Suppose that the *i*-th input attribute of an upper-level node corresponds to the output of a lower-level node, with a static attribute weight $\delta_{i}$ and a reliability coefficient $\rho_{i}$. The dynamic attribute weight of the *i*-th input under the current sample is defined as follows:

(16) $${\tilde{\delta }}_{i}=\frac{\delta_{i}\rho_{i}}{\max_{s}\left(\delta_{s}\rho_{s}\right)}$$

According to Equation (16), the effective attribute weight of the parent node depends jointly on the static importance $\delta_{i}$ learned during training and the sample-dependent reliability coefficient $\rho_{i}$ of the corresponding child node. When the child-node output is relatively dispersed, its entropy is higher and its reliability coefficient is correspondingly lower. Therefore, its relative influence on the parent-node inference is attenuated. Conversely, when the child-node output is more concentrated, its reliability coefficient is higher and the corresponding child node exerts a stronger relative influence on the parent-node inference.

When the lower-level output approaches a uniform belief distribution, its reliability coefficient decreases to 0.5 rather than zero. Consequently, the corresponding input retains part of its contribution to the upper-level inference instead of being completely eliminated. This bounded reliability mapping avoids the zero-weight problem caused by maximum-entropy outputs and maintains the continuity of the interlayer inference process.

Therefore, the proposed interlayer mechanism does not transmit only a scalar utility value. Instead, the utility value represents the expected performance location of the lower-level output, while the entropy-derived reliability coefficient provides a low-dimensional description of its distribution concentration. The two quantities are jointly incorporated into the upper-level inference through the dynamic attribute weights. In this way, the model retains part of the uncertainty-related information of the lower-level output without increasing the number of upper-level rules. The inference process of the proposed IH-BRB model is shown in Figure 3.

## 4. Optimization of IH-BRB Considering Interpretability

### 4.1. Interpretability Constraints for Optimization

The initial IH-BRB model depends on expert knowledge, which makes it highly interpretable. However, the interpretability of this model may be compromised when optimization is performed via algorithms with stochastic search properties [25]. The consequent belief degrees represent the support of a rule for different output grades and are derived from the prior probabilities and conditional probabilities according to Equation (5). When the resulting belief degrees do not follow a reasonable belief distribution through optimization, the interpretability of the model could be affected. In general, there exist some typical belief distributions, including monotonically increasing, monotonically decreasing, and unimodal, while U-shaped and multimodal distributions are considered unreasonable, as shown in Figure 4.

To address this issue, this paper imposes semantic consistency constraints at two levels, namely, the conditional probability tables of the input attributes and the rule consequent belief distributions, so as to avoid semantic conflicts in which the same indicator state or the same rule implies both superior and inferior performance. First, as described in Section 3.2, the conditional probability table of the *i*-th input attribute is given by the following:

(17) $$P^{\left(i\right)}={\left[p_{j,n}^{\left(i\right)}\right]}_{J_{i}\times N},\;p_{j,n}^{\left(i\right)}=P\left(A_{i,j}|D_{n}\right)$$

The column direction in the conditional probability table shows the likelihood distribution of each input reference level when a certain output performance grade occurs. The row direction reflects the association between a specific input reference level and various output performance grades. According to the engineering law of continuous performance degradation of a LIMU, we assume that the grade whose semantics are closest to the current state has a higher probability and that the probability gradually decreases as the semantic distance increases. Suppose that the semantic centers of the *n*-th column and the *k*-th row are denoted by $c_{i,n}$ and $q_{i,k}$, respectively. The row and column directions must satisfy the following constraints:

(18) $$\left\{\begin{array}{c} \left(p_{j+1,n}^{\left(i\right)}-p_{j,n}^{\left(i\right)}\right)\left(j+0.5-c_{i,n}\right)\leq 0,\;j=1,\ldots ,J_{i}-1\\ \left(p_{j,n+1}^{\left(i\right)}-p_{j,n}^{\left(i\right)}\right)\left(n+0.5-q_{i,j}\right)\leq 0,\;n=1,\ldots ,N-1 \end{array}\right.$$

Furthermore, to ensure that the probability peak shifts along the correct direction with performance degradation, the row and column semantic centers should also satisfy the following constraints:

(19) $$c_{i,1}\leq c_{i,2}\leq \cdots \leq c_{i,N},\;q_{i,1}\leq q_{i,2}\leq \cdots \leq q_{i,J_{i}}$$

These constraints imply that, as the output performance degrades from superior to inferior, the corresponding input probability peak should not shift backward toward a better reference level. Similarly, as the input indicator state gradually deteriorates, the output performance grades it primarily supports should not shift toward a better direction.

Secondly, at the rule consequent level, the consequent belief distributions generated by the Bayesian formulation should also satisfy generalized monotonicity constraints [26], namely,

(20) $$\left(n+0.5-\kappa_{r}\right)\left(\beta_{r,n+1}-\beta_{r,n}\right)\leq 0,\;n=1,2,\cdots ,N-1$$

From Equation (20), it can be seen that, when $\kappa_{r}=1$, the consequent belief distribution is monotonically decreasing; when $\kappa_{r}=N$, it is monotonically increasing; and, in other cases, the consequent belief distribution exhibits a unimodal pattern. Through the joint effect of the monotonicity constraints on the probability table degradation and the monotonic or unimodal constraints on the rule consequents, semantic drift of rules during optimization can be avoided, ensuring that the local BRB inference results remain consistent with engineering cognition.

### 4.2. Optimization Model with Interpretability Constraints

In the hierarchical model, each BRB node is interrelated with one another. Variations in the parameters of the lower-level nodes will affect the overall system evaluation results through interlayer propagation. Therefore, it is necessary to perform joint optimization of the parameters across all nodes. Let the parameter set of the *l*-th node be denoted as follows:

(21) $$\Phi_{l}=\left\{\theta_{l,r},\delta_{l,i},\pi_{l,n},p_{j,n,l}^{\left(i\right)}\right\},\;\Phi =\overset{N_{B}}{\underset{l=1}{⋃}}\Phi_{l}$$

where $\theta_{l,r}$, $\delta_{l,i}$, $\pi_{l,n}$ and $p_{j,n,l}^{\left(i\right)}$ denote the rule weight, attribute weight, output prior probability, and attribute conditional probability, respectively. Taking the cross-entropy between the top-level output belief distribution and the target labels as the objective function, the interpretability-constrained optimization model is formulated as follows:

(22) $$\begin{array}{cc} \min_{\Phi }\; & L\left(\Phi \right)=-\frac{1}{H}\sum_{h=1}^{H}\sum_{n=1}^{N}y_{n}^{h}\log_{2}{\hat{\beta }}_{n}^{h}\;\mathrm{where}\;y_{n}^{h}=\left\{\begin{array}{cc} 0, & n\neq n_{h}\\ 1, & n=n_{h} \end{array}\right.\\ s.t.\; & \left(p_{j+1,n,l}^{\left(i\right)}-p_{j,n,l}^{\left(i\right)}\right)\left(j+0.5-c_{l,i,n}\right)\leq 0,\\ \; & \left(p_{j,n+1,l}^{\left(i\right)}-p_{j,n,l}^{\left(i\right)}\right)\left(n+0.5-q_{l,i,j}\right)\leq 0,\\ \; & c_{l,i,1}\leq \cdots \leq c_{l,i,N_{l}},\;q_{l,i,1}\leq \cdots \leq q_{l,i,J_{l,i}},\;\left(\beta_{l,r,n+1}-\beta_{l,r,n}\right)\left(n+0.5-\kappa_{l,r}\right)\leq 0,\\ \; & 0\leq \theta_{l,r}\leq 1,\;0\leq \delta_{l,i}\leq 1,\;0\leq \pi_{l,n},\;\sum_{n=1}^{N}\pi_{l,n}=1,\;0\leq p_{j,n,l}^{\left(i\right)},\;\sum_{j=1}^{J_{i}}p_{j,n,l}^{\left(i\right)}=1. \end{array}$$

where *H* represents the number of samples; ${\hat{\beta }}_{n}^{h}$ represents the belief degree assigned to the *n*-th evaluation grade for the *h*-th sample; $n_{h}$ denotes the target performance-grade index of the *h*-th sample; and $\kappa_{l,r}$ represents the semantic center of the rule’s consequent. The above constraints ensure that both the rows and columns of the probability table and the rule consequents exhibit generalized monotonic distributions, and that the probability peaks shift in an orderly manner with performance degradation, thereby restricting the parameter search within a semantically feasible region consistent with LIMU engineering cognition.

Parameter optimization of the BRB model exhibits high-dimensional, nonlinear, and non-convex characteristics. Conventional gradient-based optimization methods are susceptible to initial values and prone to getting trapped in local optima. Recent studies have demonstrated that the projection covariance matrix adaptation evolution strategy (P-CMA-ES) can effectively solve such constrained parameter optimization problems [27]. This algorithm employs covariance matrix adaptation to learn the correlation structure and favorable search directions among parameters, and utilizes projection operators to map candidate solutions onto the feasible region, thereby balancing global search capability and constraint-handling efficiency. Therefore, this paper adopts the P-CMA-ES to optimize the parameters of the hierarchical BRB model.

In summary, the overall framework of the proposed interpretable hierarchical belief rule base based LIMU performance evaluation model is illustrated in Figure 5 below.

## 5. Experiments and Validation

### 5.1. Experimental Background

Six sets of LIMUs of the same model are taken as experimental subjects for data acquisition in this study. The experimental data were derived from monthly inspections, yielding a total of 720 valid samples. Each sample contains 24 underlying performance indicators, including bias stability, scale factor stability, and two orthogonal installation errors for each of the three-axis laser gyroscopes and three-axis quartz accelerometers. Data acquisition was conducted under ambient conditions at 24 ± 2 °C, with calibration performed using both rate turntable calibration and static calibration methods. Through consultation with domain experts and consideration of the application requirements of LIMUs in practical navigation scenarios, the overall LIMU performance was divided into four grades: Excellent (E), Good (G), Medium (M), and Poor (P), with corresponding quantitative utility values of 1, 2, 3, and 4, respectively. A smaller utility value indicates better LIMU performance, whereas a larger value indicates a higher degree of performance degradation. The target performance grade of each sample was independently assessed by five domain experts with experience in LIMU testing and performance evaluation. Each expert evaluated the samples according to the historical distributions of the 24 underlying performance indicators, equipment-use specifications, and engineering experience. Expert qualification scores were determined based on relevant working experience, performance-testing experience, and evaluation experience, and were normalized to obtain the expert weights. The four performance grades E, G, M, and P were assigned utility values of 1, 2, 3, and 4, respectively. The individual assessments were aggregated using a weighted utility score, and the final target grade was determined according to the corresponding grade intervals. The resulting expert-defined grades were used as the target labels for model training and evaluation. The final dataset contained 223 Excellent samples, 191 Good samples, 159 Medium samples, and 147 Poor samples. The subsequent evaluation therefore focuses on the consistency between the model outputs and the expert grading criterion (Figure 6).

To quantitatively evaluate this consistency, the mean squared error (MSE) and mean absolute error (MAE) are adopted as evaluation metrics, which are calculated as follows:

(23) $$\mathrm{MSE}=\frac{1}{H}\sum_{s=1}^{H}{\left({\hat{U}}_{s}-U_{s}\right)}^{2},\;\mathrm{MAE}=\frac{1}{H}\sum_{s=1}^{H}\left|{\hat{U}}_{s}-U_{s}\right|$$

where *H* denotes the number of test samples; ${\hat{U}}_{s}$ represents the predicted utility value; and $U_{s}$ represents the target utility value. The MSE measures the squared deviation between the predicted and target utility values, and is more sensitive to relatively large prediction errors. The MAE measures the mean absolute deviation between the predicted and target utility values. Smaller MSE and MAE values indicate a higher degree of agreement between the model outputs and the target performance grades.

### 5.2. Initial IH-BRB Model and Optimization

The initial hierarchical model is constructed according to the five-level physical structure established in Section 3. Taking the $G_{x}$-axis laser gyroscope as an example, its underlying inputs include bias stability *B*, scale factor stability *K*, and two installation errors *M*. Each input attribute is assigned three reference levels, namely Small (S), Medium (Me), and Large (L), with the reference values determined by domain experts based on historical experience, as shown in Table 3.

Taking the performance BRB of the $G_{x}$-axis laser gyroscope as a representative example, the node contains three input attributes, namely, the bias stability $B_{G_{x}}$, the scale factor stability $K_{G_{x}}$, and the installation error evaluation result $D(M_{G_{x}})$. The model parameters are initialized based on expert knowledge. The rule weights and static attribute weights are uniformly initialized as 1, while the prior probabilities of the four output performance grades are initialized as (0.25, 0.25, 0.25, 0.25). The initial conditional probability tables are specified by multiple domain experts according to engineering experience, as listed in Table 4. According to the Bayesian rule-generation mechanism in Equation (5), the consequent belief distribution of each rule is generated from the prior probabilities and the corresponding conditional probabilities rather than being independently assigned.

The experiments were conducted using leave-one-unit-out (LOUO) validation. In each fold, all 120 samples from one LIMU were used as the test set, while the 600 samples from the remaining five LIMUs were used for model training and parameter optimization. The procedure was repeated six times so that each LIMU served once as the test unit, and the model parameters were independently re-optimized using only the corresponding training data in each fold. Taking the $G_{x}$-axis laser gyroscope performance BRB as an example, the optimized prior probability distribution was (0.33, 0.30, 0.18, 0.19). The optimized attribute conditional probability table is shown in Table 5. The complete optimized rule base is presented in Table 6 and the LOUO evaluation results of the optimized IH-BRB model are shown in Figure 7.

Figure 7a compares the target utility values with the predictions of the initial and optimized IH-BRB models on the test set. The black hollow circles represent the target utility values, while the blue squares and orange triangles denote the predictions of the initial and optimized models, respectively. It can be observed that the predictions of the initial model show relatively large deviations from the target values for a number of samples, particularly for samples located near different performance-grade regions. After optimization, the predicted utility values are generally closer to the corresponding target values, indicating an improved agreement between the model outputs and the target performance grades. Specifically, the MSE decreases from 0.1473 to 0.0626, corresponding to a reduction of approximately 57.5%, while the MAE decreases from 0.3254 to 0.1410, representing a reduction of approximately 56.7%. These results indicate that the parameter optimization process improves the agreement between the IH-BRB outputs and the target performance grades.

Figure 7b further presents the sample-wise absolute errors before and after optimization. The blue squares and orange triangles represent the absolute errors of the initial and optimized models, respectively, while the blue dashed line and orange solid line denote their moving-average errors with a window size of 9. Compared with the initial model, the absolute errors of the optimized model are more concentrated in the lower-error region, and its moving-average error remains below that of the initial model for most test samples. Although several samples still exhibit relatively large errors, the overall error level and local error fluctuations are reduced after optimization, further confirming the effectiveness of the parameter optimization process.

### 5.3. Interpretability Analysis of the Model

#### 5.3.1. Interpretability Analysis of the Hierarchical Structure

To illustrate the interpretability of the hierarchical structure by intuitively demonstrating the layer-wise inference mechanism of the model, a sample is randomly selected from the sample set for a complete inference analysis. The overall inference process is shown in Figure 8.

As shown in Figure 8, each local node can provide a performance state with clear physical meaning. At the installation error layer, evident state differences can already be observed among different sensitive axes. Taking the $M_{G_{x}}$ node as an example, its belief distribution is [0.50, 0.24, 0.17, 0.09], with a utility value of 1.85 and an output reliability of only 0.535. Although the maximum belief degree corresponds to the Excellent grade, the relatively dispersed belief distribution indicates that the node still contains considerable uncertainty across multiple performance grades. Therefore, relying only on the grade associated with the maximum belief degree cannot fully characterize the node state. Introducing output reliability together with the utility value during interlayer propagation can further reflect the certainty of the lower-layer inference result, which also provides the basis for dynamically adjusting the effective attribute weights of the upper-layer nodes according to reliability.

At the axial-level comprehensive layer, the output of each installation error BRB is combined with the bias stability and scale factor stability of the corresponding axis to obtain the comprehensive performance evaluation result of each sensitive axis. As shown in Figure 8, the $G_{z}$ and $A_{x}$ axes provide relatively high support for the Medium grade, with maximum belief degrees of 0.85 and 0.65, respectively. Their corresponding utility values are 2.85 and 2.51, indicating that these two sensitive axes exhibit relatively weaker performance, whereas the evaluation results of the remaining axes are generally closer to the Good grade.

As the axis-level information is further propagated upward, the output belief distribution of the gyroscope subsystem is [0.02, 0.16, 0.77, 0.05], with a utility value of 2.85, indicating that its evaluation result is mainly concentrated on the Medium grade. In comparison, the accelerometer subsystem yields a belief distribution of [0.14, 0.80, 0.06, 0.00] and a utility value of 1.92, with its performance state mainly concentrated on the Good grade. This indicates that, for the current sample, the overall performance of the gyroscope subsystem is relatively weaker than that of the accelerometer subsystem. After further fusing the states of the two subsystems at the overall decision-making layer, the final LIMU belief distribution is obtained as [0.00, 0.07, 0.90, 0.03], with a utility value of 2.96, and the final evaluation result is therefore clearly concentrated on the Medium grade.

This result, to a certain extent, demonstrates the rationality of the hierarchical fusion mechanism of the IH-BRB model and provides a basis for layer-by-layer interpretation and traceability of the final system-level evaluation result.

#### 5.3.2. Local Semantic Interpretability Analysis

To examine the semantic validity of local rules, the BRB node corresponding to the installation error performance state of the Gx axis is selected as an example. The rule consequent belief distributions obtained from the traditional unconstrained optimization model and the proposed constrained optimization model are compared. Both models adopt the same initial parameters, training data, and optimization iterations, with the sole difference being that the proposed model imposes interpretability constraints on the conditional probability tables and rule consequents. The rule consequent belief distributions of the BRB node for the Gx installation error performance state are shown in Figure 9.

From Figure 9, it can be observed that, as the two installation error stability indicators $M_{Gxy}$ and $M_{Gxz}$ gradually deteriorate from S to Me and L, the consequent belief distributions of the IH-BRB exhibit an overall orderly transition from E toward G, M, and P. When either installation error indicator is fixed, the probability mass of the rule consequent generally shifts toward poorer performance grades as the other indicator deteriorates. This indicates that the interpretability constraints are capable of maintaining semantic consistency between the antecedent states and the corresponding consequent performance grades.

For Rules 1 and 9, the dominant semantic centers of the two models are largely consistent, mainly supporting E and P, respectively, indicating that the interpretability constraints do not unnecessarily modify boundary rules that are already consistent with engineering cognition. Rules 2, 4, and 7 still distribute their belief degrees mainly over adjacent performance grades, showing that the constrained model preserves the capability of BRB to represent uncertainty in grade-transition regions.

For Rule 6, the unconstrained model assigns relatively large belief degrees to both G and P, while the belief degree assigned to the intermediate grade M is considerably lower, resulting in a non-adjacent multimodal distribution. After the interpretability constraints are imposed, the probability mass is redistributed and mainly concentrated on M, while decreasing smoothly toward the adjacent grades G and P, thereby eliminating the local semantic conflict.

In summary, the interpretability constraints preserve the uncertainty representation of BRB while eliminating unreasonable consequent distributions, thereby maintaining semantically consistent degradation trends and improving the interpretability of the optimized rule base.

### 5.4. Comparative Experiments

The IH-BRB is an interpretable evaluation model constructed by introducing a hierarchical structure, interpretability constraints, and an interlayer reliability propagation mechanism into the traditional BRB framework. The interpretability constraints restrict the feasible region of model parameters and may affect the fitting flexibility of the model. To evaluate the model performance and examine the influence of these constraints, comparative experiments were conducted using the proposed interpretable hierarchical Bayesian belief rule base (IH-BRB), hierarchical Bayesian belief rule base without interpretability constraints (H-BRB), disjunctive belief rule base (D-BRB), K-nearest neighbors (KNN), support vector machine (SVM), random forest (RF), and backpropagation neural network (BPNN). All models were evaluated using the same six-fold leave-one-unit-out validation scheme. In each fold, one LIMU was used as the test unit and the remaining five LIMUs were used for model training. Model parameters were independently trained or optimized using only the corresponding training data. H-BRB retains the same hierarchical structure and inference mechanism as IH-BRB but removes the interpretability constraints, providing a direct comparison for examining their influence. The mean and standard deviation of the evaluation metrics across the six held-out LIMUs were used for model comparison. The experimental results are presented in Figure 10.

As shown in Figure 10, the seven models were evaluated using six-fold LOUO validation. The IH-BRB model achieves an average MSE of 0.0635±0.0063 and an average MAE of 0.1255±0.0140. Among all the compared methods, H-BRB obtains the lowest average MSE and MAE, with values of 0.0503±0.0118 and 0.1039±0.0181, respectively. The corresponding MAE and MSE results for each fold are presented in Figure 10a and Figure 10b, respectively, and the average results with standard deviations are summarized in Figure 10c.

Compared with IH-BRB, H-BRB achieves lower MSE and MAE under the current experimental setting, indicating higher evaluation accuracy. This difference is consistent with the greater fitting flexibility of H-BRB, whereas the interpretability constraints imposed in IH-BRB restrict the feasible parameter space during optimization. Meanwhile, these constraints preserve the semantic consistency of the optimized parameters and rule consequents, reflecting a trade-off between the evaluation performance and engineering interpretability.

Compared with D-BRB, IH-BRB reduces the average MSE from 0.2604 to 0.0635 and the average MAE from 0.3617 to 0.1255, corresponding to reductions of approximately 75.6% and 65.3%, respectively. The considerably larger errors of D-BRB indicate that, under the current dataset and parameter settings, the hierarchical representation adopted by IH-BRB is more effective in organizing the multi-source performance information of the LIMU.

Compared with the data-driven methods, IH-BRB achieves lower average MSE values than KNN, SVM, RF, and BPNN, with reductions of approximately 8.1%, 61.8%, 10.6%, and 25.6%, respectively. In terms of MAE, IH-BRB achieves lower values than SVM, RF, and BPNN, with reductions of approximately 42.6%, 30.9%, and 32.1%, respectively, while its MAE is close to that of KNN. In addition to evaluation performance, IH-BRB provides layer-wise explanatory information through rule activation, belief distributions, utility values, and reliability coefficients, enabling the final evaluation result to be traced back to the subsystem, sensitive axis, and underlying indicator levels.

Overall, IH-BRB preserves rule-semantic consistency and hierarchical traceability while maintaining a competitive evaluation performance, indicating that the proposed model achieves a reasonable balance between the evaluation performance and engineering interpretability.

## 6. Conclusions

This paper proposes a hierarchical interpretable Bayesian belief rule base performance evaluation model for LIMU to address the challenges of small sample sizes, high-dimensional indicators, state uncertainty, and difficulty in interpreting evaluation results during long-term storage and service. The main contributions are summarized as follows:

(1) A five-level hierarchical evaluation model consistent with the physical structure of the LIMU is constructed. Based on the physical composition and error propagation paths of the LIMU, the 24 underlying indicators are organized into a five-level structure, namely, “indicator input—installation error—single-axis performance—device subsystem—overall system performance,” such that each node at every level corresponds to a clear engineering entity, thereby reducing the rule scale while enabling layer-by-layer traceability of the evaluation process.

(2) An interlayer propagation mechanism that balances information compression and uncertainty preservation is designed. The model employs utility values to represent the performance level of lower-level nodes, constructs reliability measures using normalized information entropy, and dynamically adjusts the upper-level attribute weights accordingly, thus achieving a trade-off between information compression and uncertainty retention.

(3) Interpretability constraints for optimization are established. Semantic consistency constraints are imposed at two levels, namely, the attribute conditional probability tables and the rule consequent belief distributions, and the P-CMA-ES algorithm is employed for joint optimization of model parameters to suppress multimodal distributions, grade reversals, and rule semantic drift.

Nevertheless, the following limitations still exist in this study:

(1) The current validation uses expert-defined target grades derived from the 24 performance indicators, and the reported errors therefore characterize consistency with the expert grading criterion. Future work will extend the validation using independently obtained performance or degradation information.

(2) The Bayesian rule consequent generation in Equation (5) assumes conditional independence among antecedent attributes within each local BRB node given the output grade. This approximation reduces parameterization complexity without implying global independence, as cross-axis and subsystem coupling is represented by the hierarchical structure. Residual within-node dependence remains a limitation and will be addressed using joint conditional or other dependence models in future work.

(3) The current model employs a triangular linear membership function for mapping continuous indicators to reference levels, which assumes a fixed linear transition between adjacent reference levels. To a certain extent, this approach does not account for the non-uniform distribution of indicator samples or the influence of measurement noise and other uncertain factors on grade boundaries, which may cause the matching degrees of certain indicators to deviate from the actual data distribution.

## Figures and Tables

**Figure 1:**
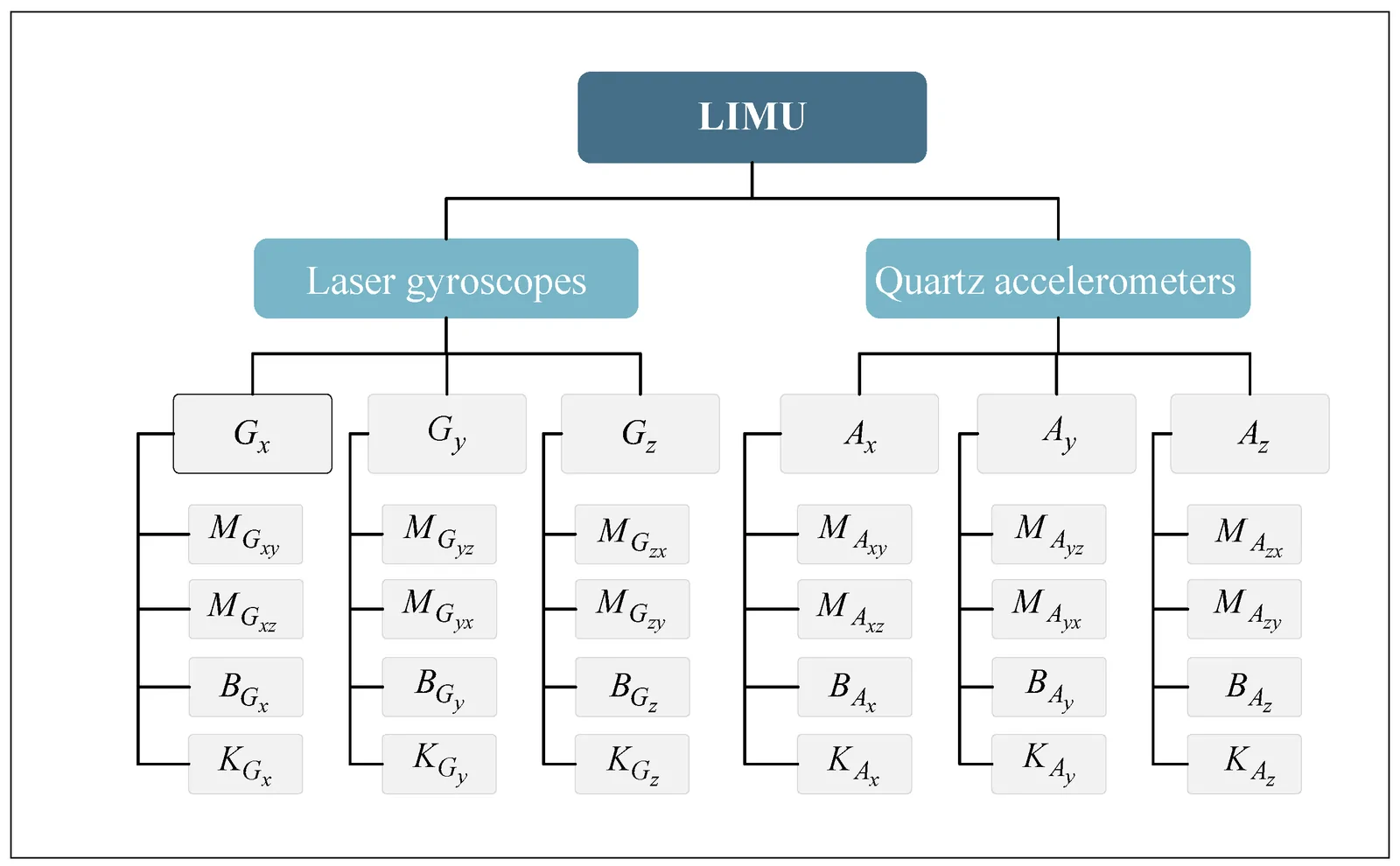
Physical organization of the 24-dimensional input vector in Equation (4).

**Figure 2:**
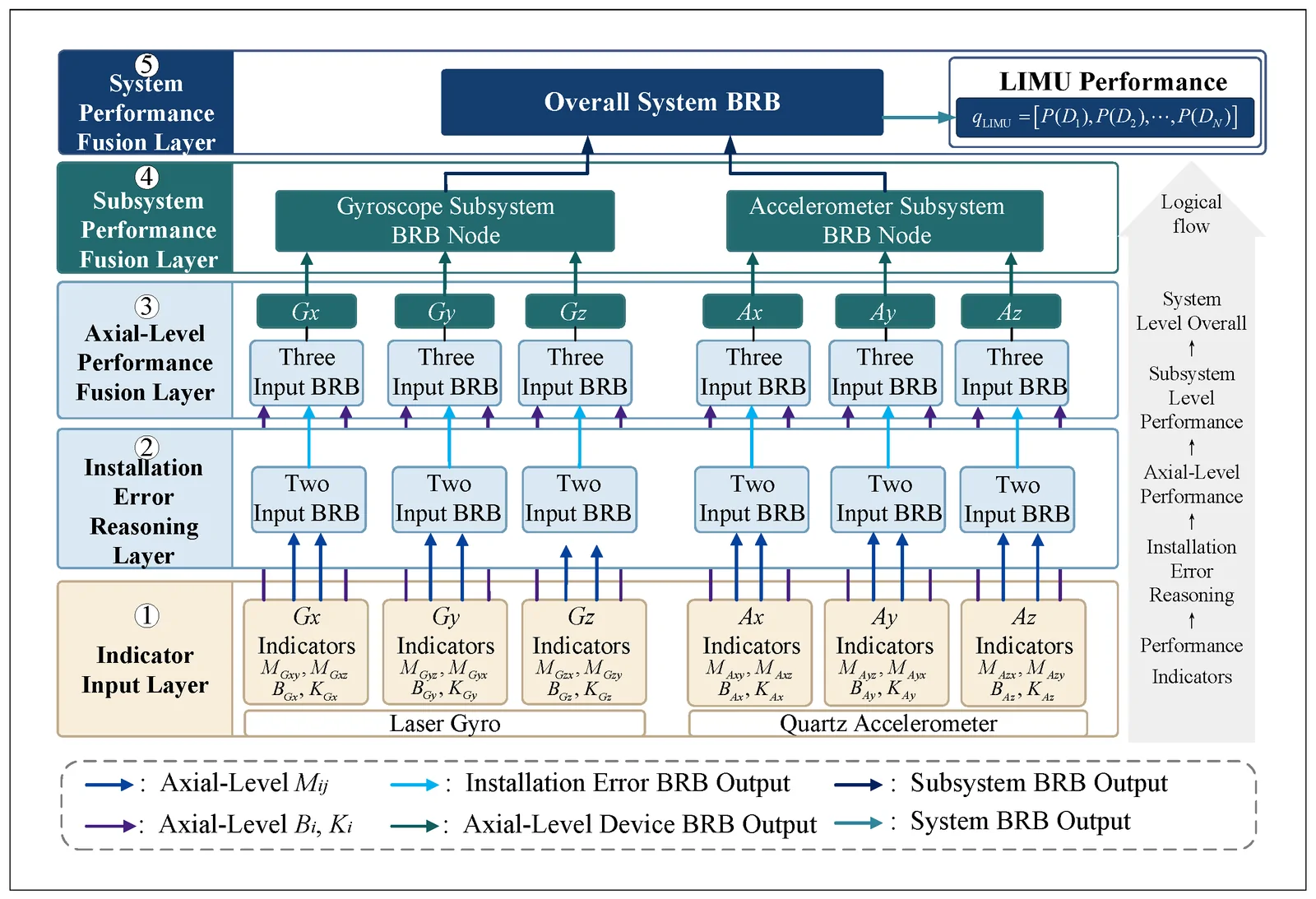
Hierarchical architecture of the IH-BRB model.

**Figure 3:**
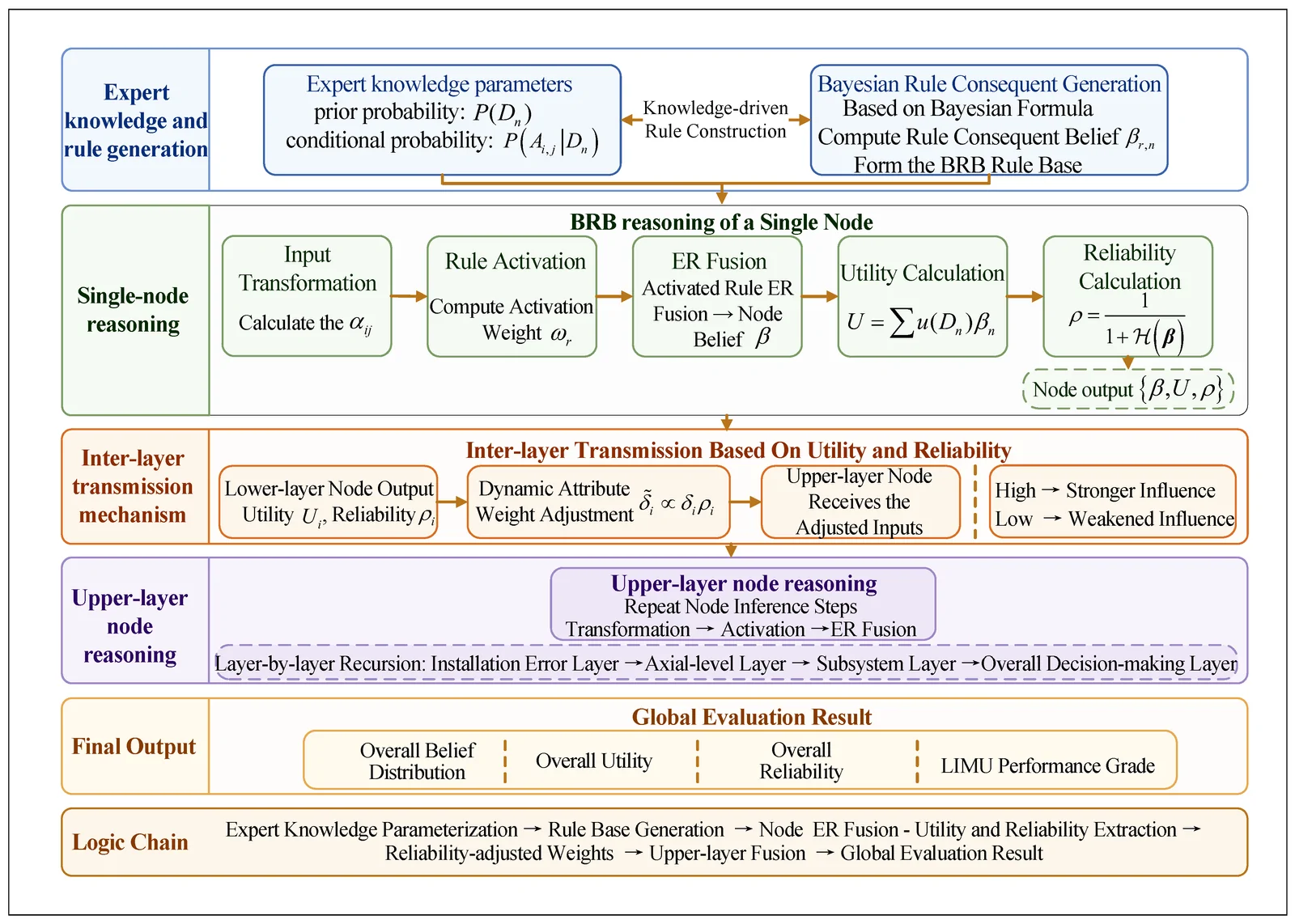
IH-BRB inference framework.

**Figure 4:**
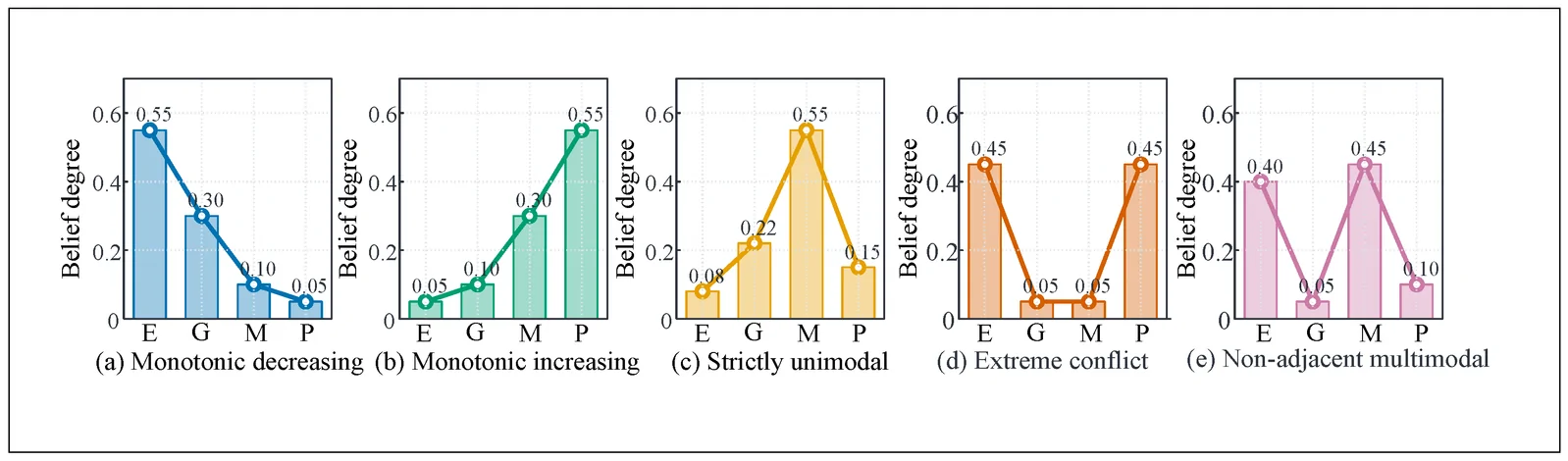
Different types of belief distributions.

**Figure 5:**
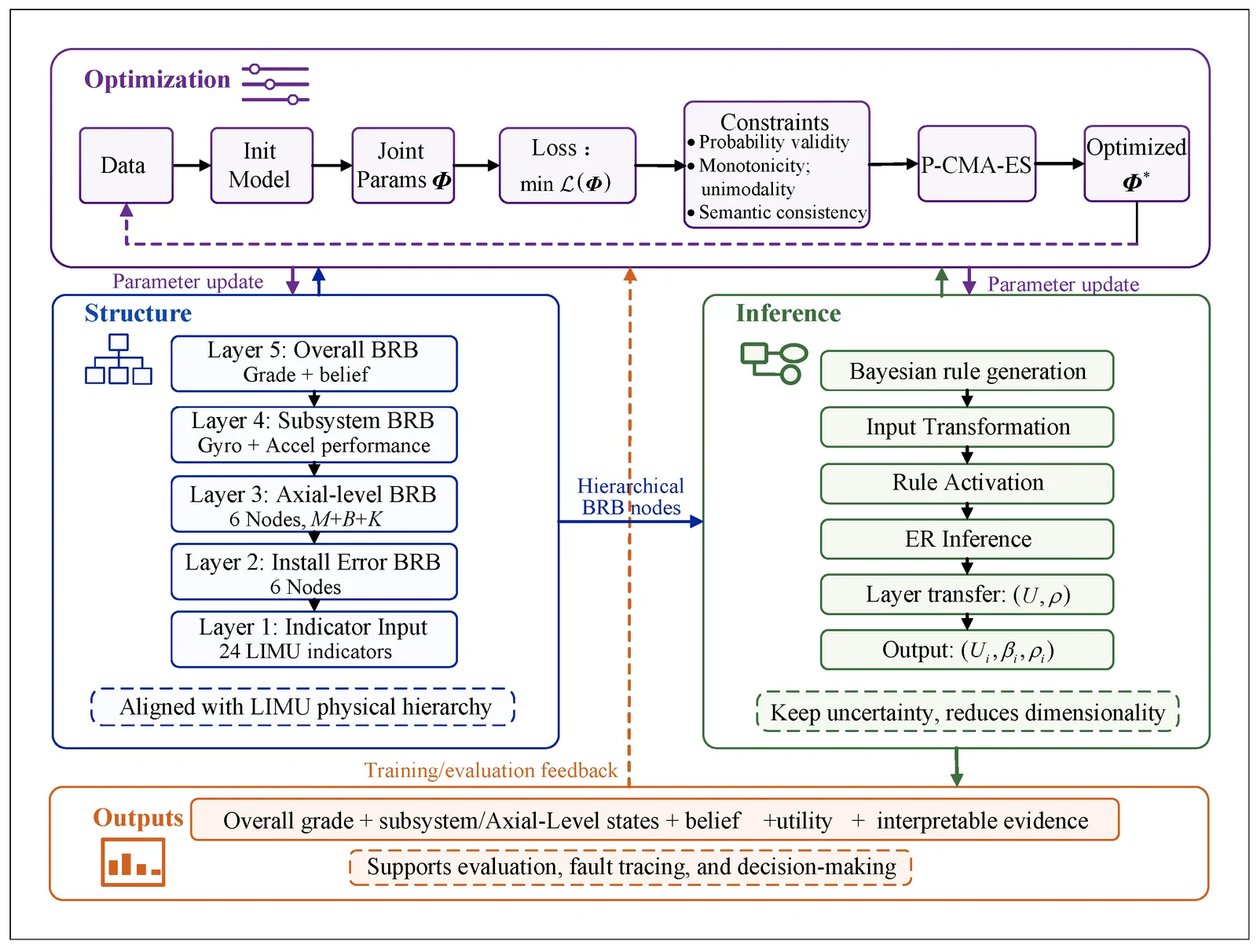
Overall framework of the IH-BRB based LIMU performance evaluation model.

**Figure 6:**
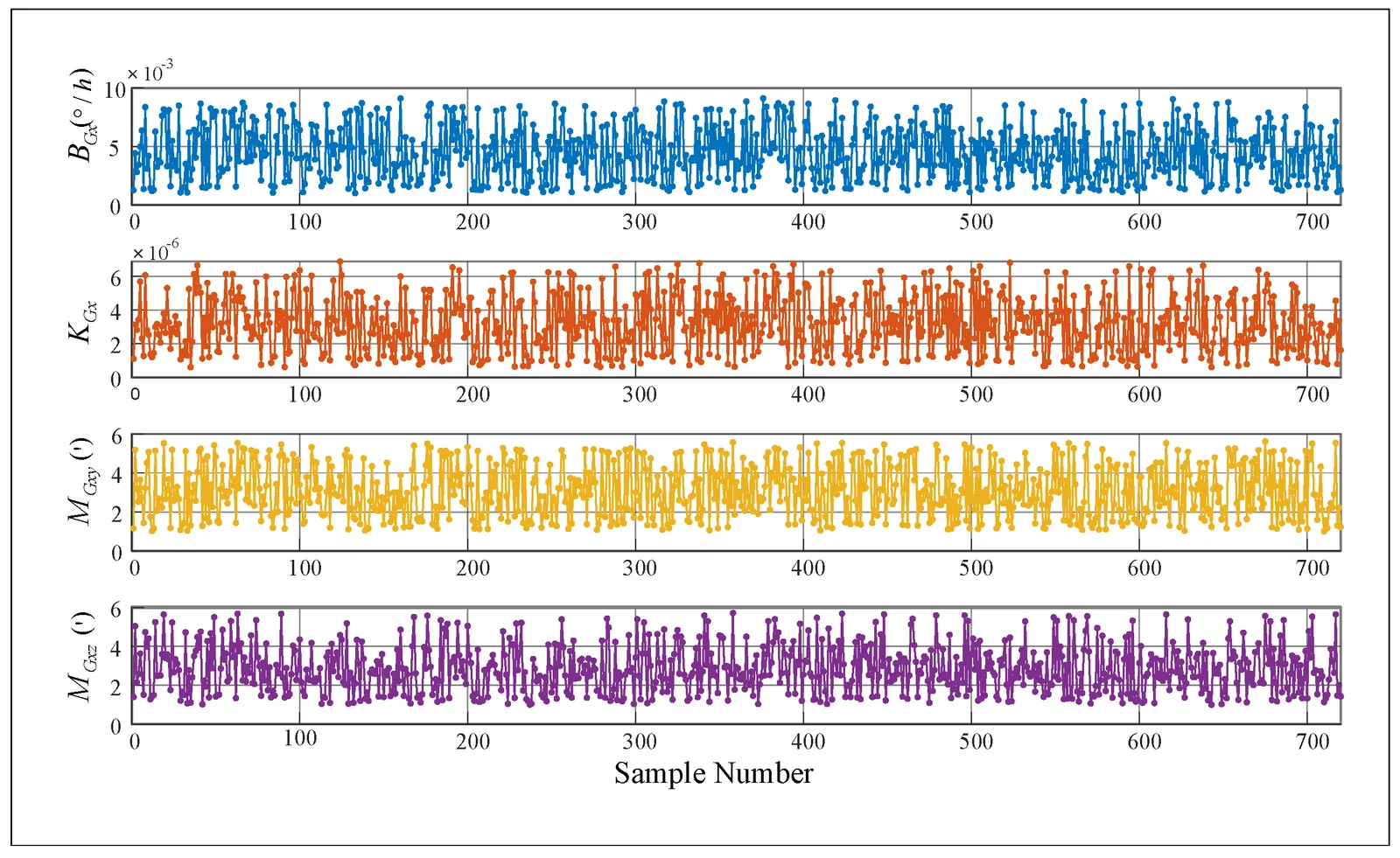
Raw data of the Gx gyroscope.

**Figure 7:**
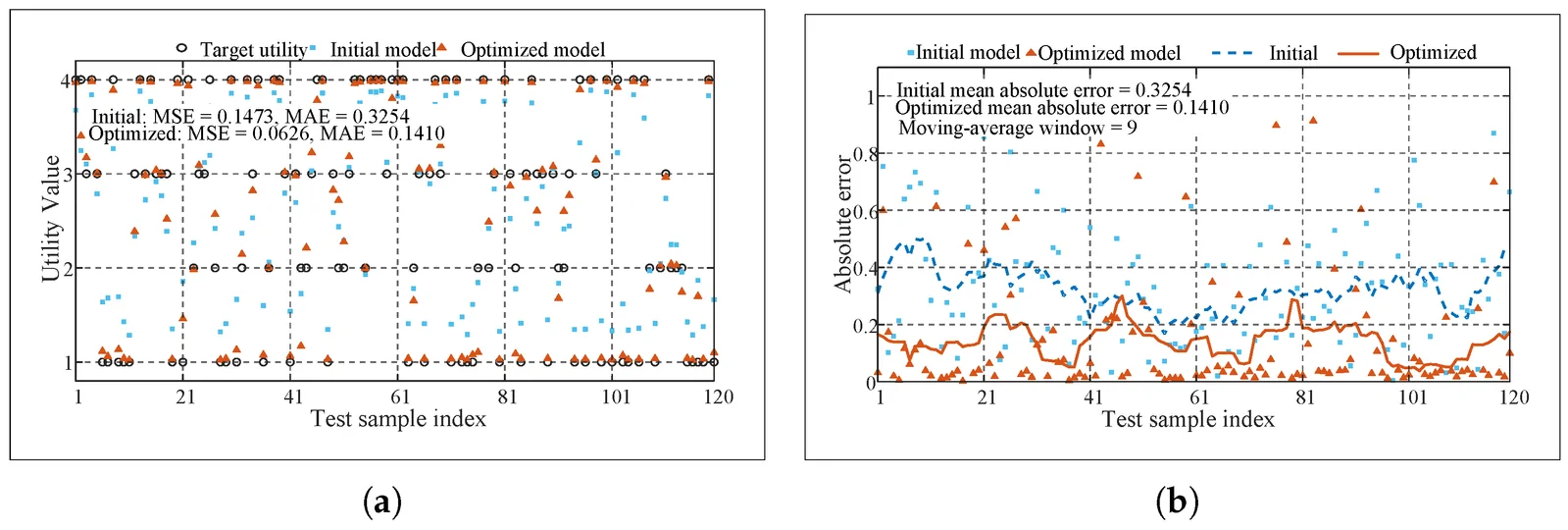
Comparison of test results of the IH-BRB model before and after optimization; (**a**) test set utility comparison: (**b**) test set absolute error.

**Figure 8:**
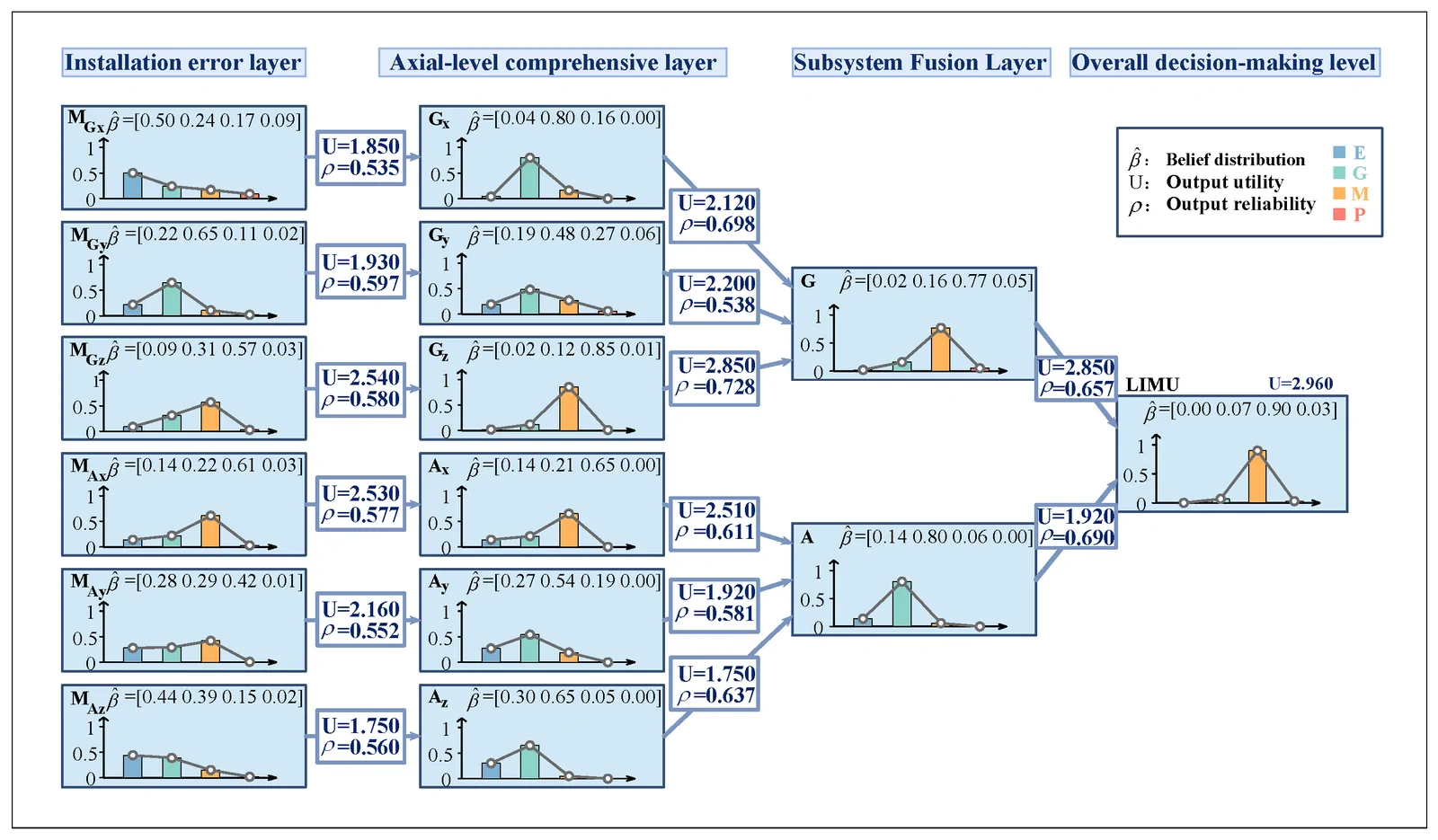
Schematic diagram of single-sample inference.

**Figure 9:**
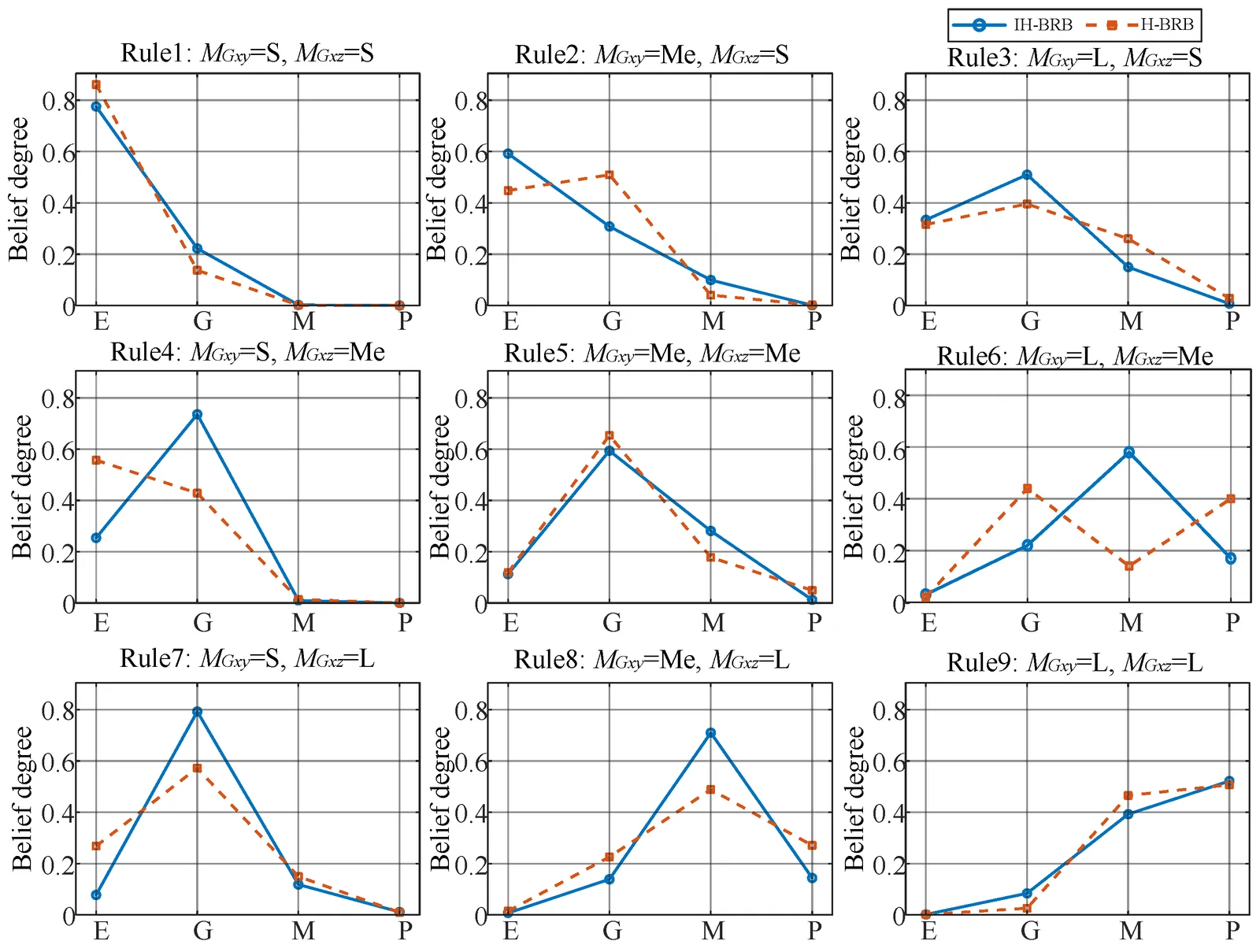
Comparison of rule consequent belief distributions.

**Figure 10:**
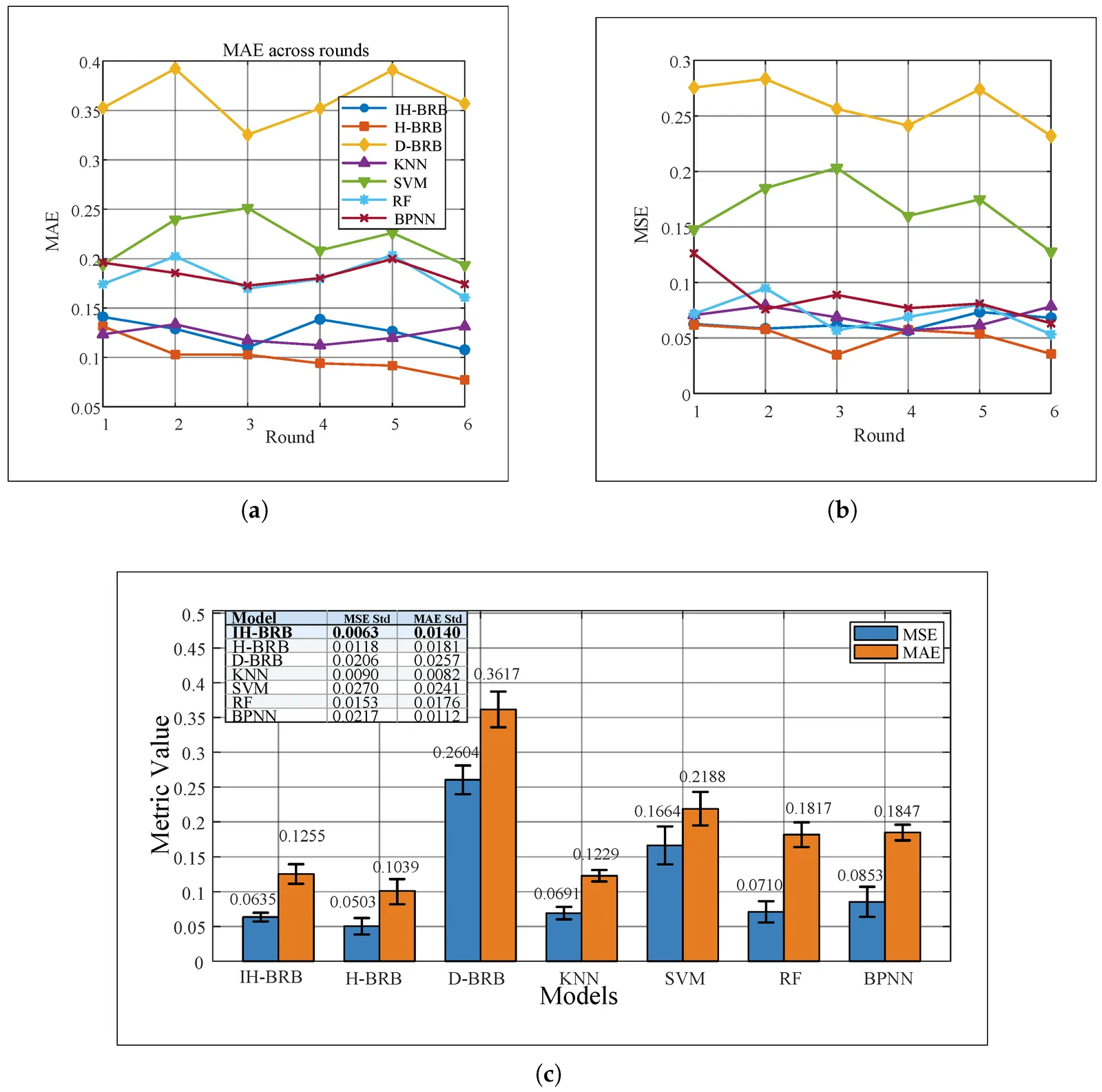
Comparison of experimental results among different evaluation models: (**a**) MAE across rounds; (**b**) MSE across rounds; (**c**) the mean and standard deviation of MSE and MAE.

**Table 1:** Complete rule base of the $G_{x}$-axis installation error BRB node.

Rule	$M_{\mathrm{Gxy}}$	$M_{\mathrm{Gxz}}$	Consequent Belief Distribution	Rule Weight
$R_{1}$	S	S	$\left\{\left(E,\beta_{1,1}\right),\left(G,\beta_{1,2}\right),\left(M,\beta_{1,3}\right),\left(P,\beta_{1,4}\right)\right\}$	$\theta_{1}$
$R_{2}$	Me	S	$\left\{\left(E,\beta_{2,1}\right),\left(G,\beta_{2,2}\right),\left(M,\beta_{2,3}\right),\left(P,\beta_{2,4}\right)\right\}$	$\theta_{2}$
$R_{3}$	L	S	$\left\{\left(E,\beta_{3,1}\right),\left(G,\beta_{3,2}\right),\left(M,\beta_{3,3}\right),\left(P,\beta_{3,4}\right)\right\}$	$\theta_{3}$
$R_{4}$	S	Me	$\left\{\left(E,\beta_{4,1}\right),\left(G,\beta_{4,2}\right),\left(M,\beta_{4,3}\right),\left(P,\beta_{4,4}\right)\right\}$	$\theta_{4}$
$R_{5}$	Me	Me	$\left\{\left(E,\beta_{5,1}\right),\left(G,\beta_{5,2}\right),\left(M,\beta_{5,3}\right),\left(P,\beta_{5,4}\right)\right\}$	$\theta_{5}$
$R_{6}$	L	Me	$\left\{\left(E,\beta_{6,1}\right),\left(G,\beta_{6,2}\right),\left(M,\beta_{6,3}\right),\left(P,\beta_{6,4}\right)\right\}$	$\theta_{6}$
$R_{7}$	S	L	$\left\{\left(E,\beta_{7,1}\right),\left(G,\beta_{7,2}\right),\left(M,\beta_{7,3}\right),\left(P,\beta_{7,4}\right)\right\}$	$\theta_{7}$
$R_{8}$	Me	L	$\left\{\left(E,\beta_{8,1}\right),\left(G,\beta_{8,2}\right),\left(M,\beta_{8,3}\right),\left(P,\beta_{8,4}\right)\right\}$	$\theta_{8}$
$R_{9}$	L	L	$\left\{\left(E,\beta_{9,1}\right),\left(G,\beta_{9,2}\right),\left(M,\beta_{9,3}\right),\left(P,\beta_{9,4}\right)\right\}$	$\theta_{9}$

**Table 2:** Probability parameter table for a BRB node.

Indicator	$x_{1}$	$x_{2}$	⋯	$x_{d}$
Reference	$\left\{A_{1,1},A_{1,2},\ldots ,A_{1,J_{1}}\right\}$	$\left\{A_{2,1},A_{2,2},\ldots ,A_{2,J_{2}}\right\}$	⋯	$\left\{A_{d,1},A_{d,2},\ldots ,A_{d,J_{d}}\right\}$
$D_{1}$	$\left\{p_{1,1}^{\left(1\right)},p_{2,1}^{\left(1\right)},\ldots ,p_{J_{1},1}^{\left(1\right)}\right\}$	$\left\{p_{1,1}^{\left(2\right)},p_{2,1}^{\left(2\right)},\ldots ,p_{J_{2},1}^{\left(2\right)}\right\}$	⋯	$\left\{p_{1,1}^{\left(d\right)},p_{2,1}^{\left(d\right)},\ldots ,p_{J_{d},1}^{\left(d\right)}\right\}$
$D_{2}$	$\left\{p_{1,2}^{\left(1\right)},p_{2,2}^{\left(1\right)},\ldots ,p_{J_{1},2}^{\left(1\right)}\right\}$	$\left\{p_{1,2}^{\left(2\right)},p_{2,2}^{\left(2\right)},\ldots ,p_{J_{2},2}^{\left(2\right)}\right\}$	⋯	$\left\{p_{1,2}^{\left(d\right)},p_{2,2}^{\left(d\right)},\ldots ,p_{J_{d},2}^{\left(d\right)}\right\}$
⋯	⋯	⋯	⋯	⋯
$D_{N}$	$\left\{p_{1,N}^{\left(1\right)},p_{2,N}^{\left(1\right)},\ldots ,p_{J_{1},N}^{\left(1\right)}\right\}$	$\left\{p_{1,N}^{\left(2\right)},p_{2,N}^{\left(2\right)},\ldots ,p_{J_{2},N}^{\left(2\right)}\right\}$	…	$\left\{p_{1,N}^{\left(d\right)},p_{2,N}^{\left(d\right)},\ldots ,p_{J_{d},N}^{\left(d\right)}\right\}$

**Table 3:** Performance indicator grades and reference values of inertial instruments.

Inertial Instrument	Indicator	Reference Level	Reference Value
	*B*	(S, Me, L)	$\left(1.0,4.5,8.0\right)\times 10^{-3}$ °/h
Laser gyroscope	*K*	(S, Me, L)	$\left(0.6,3.4,6.2\right)\times 10^{-6}$
	*M*	(S, Me, L)	${\left(1.0,3.0,5.0\right)}^{\prime }$
	*B*	(S, Me, L)	$\left(0.8,4.0,7.2\right)\times 10^{-7}\;g_{0}$
Quartz accelerometer	*K*	(S, Me, L)	$\left(0,1.0,2.0\right)\times 10^{-5}\;g/g$
	*M*	(S, Me, L)	${\left(1.0,3.0,5.0\right)}^{\prime }$

**Table 4:** Initial conditional probability table for the $G_{x}$-axis laser gyroscope performance BRB.

Grade	$B_{G_{x}}=\left\{S,\mathrm{Me},L\right\}$	$K_{G_{x}}=\left\{S,\mathrm{Me},L\right\}$	$D\left(M_{G_{x}}\right)=\left\{E,G,M,P\right\}$
E	(0.80, 0.20, 0.00)	(0.80, 0.20, 0.00)	(0.80, 0.20, 0.00, 0.00)
G	(0.50, 0.40, 0.10)	(0.50, 0.40, 0.10)	(0.30, 0.50, 0.20, 0.00)
M	(0.20, 0.50, 0.30)	(0.20, 0.50, 0.30)	(0.10, 0.20, 0.50, 0.20)
P	(0.10, 0.30, 0.60)	(0.10, 0.30, 0.60)	(0.00, 0.10, 0.30, 0.60)

**Table 5:** Optimized conditional probability table for the $G_{x}$-axis laser gyroscope performance BRB.

Grade	$B_{G_{x}}=\left\{S,\mathrm{Me},L\right\}$	$K_{G_{x}}=\left\{S,\mathrm{Me},L\right\}$	$D\left(M_{G_{x}}\right)=\left\{E,G,M,P\right\}$
E	(0.74, 0.21, 0.05)	(0.68, 0.25, 0.07)	(0.65, 0.30, 0.04, 0.01)
G	(0.38, 0.52, 0.10)	(0.36, 0.52, 0.12)	(0.21, 0.46, 0.30, 0.03)
M	(0.13, 0.42, 0.45)	(0.20, 0.46, 0.34)	(0.04, 0.34, 0.44, 0.18)
P	(0.09, 0.28, 0.63)	(0.07, 0.22, 0.71)	(0.01, 0.07, 0.25, 0.67)

**Table 6:** Complete optimized rule base of the $G_{x}$-axis laser gyroscope performance BRB.

Rule	$B_{G_{x}}$	$K_{G_{x}}$	$D(M_{G_{x}})$	$\theta_{r}$	$\left(\beta_{r,E},\beta_{r,G},\beta_{r,M},\beta_{r,P}\right)$
$R_{1}$	S	S	E	0.8403	(0.9245, 0.0738, 0.0016, 0.0001)
$R_{2}$	S	S	G	0.9054	(0.7079, 0.2683, 0.0226, 0.0012)
$R_{3}$	S	S	M	0.9070	(0.3117, 0.5777, 0.0966, 0.0140)
$R_{4}$	S	S	P	0.9662	(0.3661, 0.2714, 0.1857, 0.1768)
$R_{5}$	S	Me	E	0.8374	(0.7544, 0.2367, 0.0082, 0.0007)
$R_{6}$	S	Me	G	0.8580	(0.3699, 0.5509, 0.0739, 0.0053)
$R_{7}$	S	Me	M	0.9222	(0.0943, 0.6866, 0.1828, 0.0363)
$R_{8}$	S	Me	P	0.9146	(0.0892, 0.2597, 0.2830, 0.3681)
$R_{9}$	S	L	E	0.9532	(0.7703, 0.1992, 0.0221, 0.0084)
$R_{10}$	S	L	G	0.9131	(0.3424, 0.4203, 0.1806, 0.0567)
$R_{11}$	S	L	M	0.9442	(0.0604, 0.3625, 0.3091, 0.2680)
$R_{12}$	S	L	P	0.9506	(0.0168, 0.0404, 0.1411, 0.8017)
$R_{13}$	Me	S	E	0.9477	(0.7113, 0.2738, 0.0140, 0.0009)
$R_{14}$	Me	S	G	0.9197	(0.3116, 0.5694, 0.1133, 0.0057)
$R_{15}$	Me	S	M	0.8608	(0.0716, 0.6402, 0.2528, 0.0354)
$R_{16}$	Me	S	P	0.9244	(0.0639, 0.2285, 0.3692, 0.3384)
$R_{17}$	Me	Me	E	0.8388	(0.3778, 0.5716, 0.0467, 0.0039)
$R_{18}$	Me	Me	G	0.9588	(0.0942, 0.6765, 0.2144, 0.0149)
$R_{19}$	Me	Me	M	0.8964	(0.0160, 0.5627, 0.3537, 0.0676)
$R_{20}$	Me	Me	P	0.9585	(0.0104, 0.1457, 0.3746, 0.4693)
$R_{21}$	Me	L	E	0.9365	(0.3714, 0.4630, 0.1211, 0.0445)
$R_{22}$	Me	L	G	0.9355	(0.0678, 0.4015, 0.4074, 0.1233)
$R_{23}$	Me	L	M	0.8557	(0.0073, 0.2114, 0.4258, 0.3555)
$R_{24}$	Me	L	P	0.8628	(0.0016, 0.0184, 0.1515, 0.8285)
$R_{25}$	L	S	E	0.9866	(0.7086, 0.2203, 0.0630, 0.0081)
$R_{26}$	L	S	G	0.9780	(0.2333, 0.3443, 0.3817, 0.0407)
$R_{27}$	L	S	M	0.9569	(0.0348, 0.2509, 0.5521, 0.1622)
$R_{28}$	L	S	P	0.9899	(0.0125, 0.0361, 0.3252, 0.6262)
$R_{29}$	L	Me	E	0.9073	(0.3477, 0.4248, 0.1933, 0.0342)
$R_{30}$	L	Me	G	0.8330	(0.0540, 0.3130, 0.5526, 0.0804)
$R_{31}$	L	Me	M	0.8779	(0.0059, 0.1682, 0.5893, 0.2366)
$R_{32}$	L	Me	P	0.9148	(0.0017, 0.0188, 0.2698, 0.7097)
$R_{33}$	L	L	E	0.8697	(0.2171, 0.2186, 0.3185, 0.2458)
$R_{34}$	L	L	G	0.8978	(0.0200, 0.0956, 0.5408, 0.3436)
$R_{35}$	L	L	M	0.9690	(0.0013, 0.0313, 0.3513, 0.6161)
$R_{36}$	L	L	P	0.9397	(0.0002, 0.0017, 0.0799, 0.9182)

## Data Availability

The datasets presented in this article are not readily available because they are subject to confidentiality restrictions. Requests to access the datasets should be directed to the corresponding author.

## Abbreviations

The following abbreviations are used in this manuscript:

AHP	Analytic hierarchy process
BPNN	Backpropagation neural network
BRB	Belief rule base
D-BRB	Disjunctive belief rule base
E/G/M/P	Excellent/Good/Medium/Poor
ER	Evidential reasoning
H-BRB	Hierarchical belief rule base without interpretability constraints
IH-BRB	Interpretable hierarchical Bayesian belief rule base
KNN	K-nearest neighbors
LIMU	Laser strapdown inertial measurement unit
MAE	Mean absolute error
MSE	Mean squared error
P-CMA-ES	Projection covariance matrix adaptation evolution strategy
RF	Random forest
S/Me/L	Small/Medium/Large
SVM	Support vector machine
LOUO	leave-one-unit-out
